# DNA methylation at the crossroads of gene and environment interactions

**DOI:** 10.1042/EBC20190031

**Published:** 2019-11-29

**Authors:** Pui-Pik Law, Michelle L. Holland

**Affiliations:** Department of Medical and Molecular Genetics, School of Basic and Medical Biosciences, King’s College London, U.K.

**Keywords:** Developmental programming, DNA methylation, Gene-environment interactions

## Abstract

DNA methylation is an epigenetic mark involved in regulating genome function and is critical for normal development in mammals. It has been observed that the developmental environment can lead to permanent changes in gene expression and DNA methylation, at least at ‘metastable epialleles’. These are defined as regions of the genome that show a variable epigenetic state that is established early in development and maintained through subsequent cell divisions. However, the majority of the known genome does not behave in this manner. Here, we use the developmental origins of adult disease hypothesis to understand environmental epigenomics. Some challenges to studying how DNA methylation is influenced by the environment include identifying DNA methylation changes associated with an environmental exposure in tissues with a complex cellular composition and at genomic regions for which DNA methylation is dynamically regulated in a cell-type specific manner. We also offer a perspective of how emerging technologies may be useful for dissecting the functional contribution of exposure-associated epigenetic changes and highlight recent evidence that suggests that genomic regions that are absent from genome assemblies may be unappreciated hotspots for environmental modulation of the epigenetic state.

## Introduction

Epigenetic mechanisms are crucial to development in multicellular organisms and act to ensure the correct temporal and spatial patterns of gene expression [1]. Although this epigenetic behaviour is genetically programmed, the inherent plasticity of these mechanisms has led to the hypothesis that environmental exposures may influence epigenetic control of gene expression and mediate the interaction of environment and genotype to determine phenotype [2].

In mammals, DNA methylation is the most frequently assayed epigenetic mark due to it being chemically stable and easy to profile. However, it is worthy to note that functional changes in the epigenome are reflected in multifactorial changes in chromatin structure, nuclear localisation and binding factors [3].

## Epialleles are barometers for environmental effects on the epigenome

**Figure 1:**
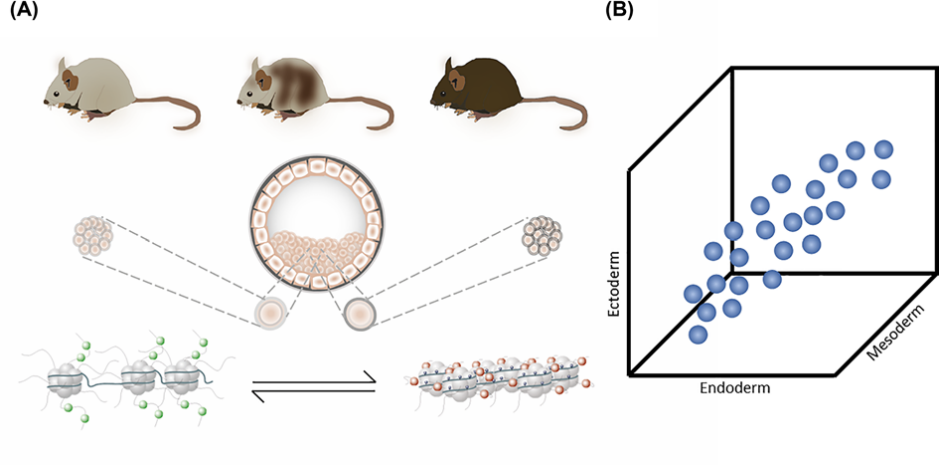
Developmental origins of metastable epialleles and how this is reflected in DNA methylation (**A**) For some parts of the genome, the decision to establish a euchromatic (left, bottom panel) or heterochromatic state (right, lower panel) is stochastic, occurs prior to germ layer specification and can vary from cell to cell. There is evidence that environmental factors can shift the balance of this decision towards one state or the other. Once the epigenetic state is established for a given cell, it is stably inherited to the mitotic progeny of that cell (middle panel). Therefore, the adult organism is an epigenetic mosaic representative of the proportion of cells that established one state or the other in early development. In the case of metastatic epialleles at the *Agouti* locus in mice, this is reflected by coat colour (top panel). Only 0.1% of the genome seems to behave in this manner. (**B**) The epigenetic state of metastable epialleles is established prior to differentiation of the three germ layers. This is reflected by covariation in the bulk methylation level of tissues derived from each germ layer (endoderm, mesoderm, ectoderm).

Metastable epialleles are genomic regions that can have a variable epigenetic state that is established stochastically in the early embryo and maintained throughout tissue differentiation [4]. Inbred mice with metastable epialleles directing an observable phenotype (e.g. coat colour for the Agouti viable yellow allele) provide exquisite examples of how epigenetic variation can contribute to phenotypic variation [5–7]. Using these classic models, it was subsequently shown that establishment of the epigenetic state at metastable epialleles could be influenced by maternal nutrition, altering the expressivity of the associated phenotypes in offspring [8,9] (Figure 1). These observations were intriguing in light of increasing evidence that the developmental environment (e.g. maternal diet and health) can influence foetal growth and the lifetime health of the offspring, a phenomenon termed ‘developmental programming’ [10].

Subsequently, a substantial effort was made to establish how pervasive epigenetic perturbations by the environment are in mammals and to what extent they contribute to phenotypic outcomes, using developmental programming as an experimental paradigm. The underlying hypothesis was that developmental processes that involve extensive epigenetic reprogramming, such as embryogenesis and gametogenesis, are particularly susceptible to environmental perturbations that may alter the epigenome, gene expression, cell function and physiology. An attractive idea has been that lasting cell-intrinsic epigenetic changes may underlie the association between developmental exposures and adult disease risk [11]. The allure of such an idea is the potential reversibility of these effects. However, although it is now well accepted that environment can impact both phenotype and the epigenome, establishing a causative role for DNA methylation in mediating the environmental impact on phenotype is more challenging and most evidence to date is associative in nature.

## Developmental programming as a model for environmentally driven epigenetic effects

The association between environmental exposure, altered DNA methylation and phenotypic consequence has been studied widely in offspring from animal models of maternal dietary intervention (e.g. protein restriction [12–17] and high-fat diet [18–21]). There are also some rare examples of comparable ‘natural experiments’ in humans, such as the offspring of pregnant women exposed to extreme caloric restriction during the Dutch Hunger Winter [22,23], or strong seasonal variation in the diet of subsistence farmers in Gambia [24].

Early studies in the field were limited to candidate gene approaches where associated phenotypes, such as hypertension induced by protein restriction during foetal and/or perinatal development [25–28], were used to investigate methylation changes at genes that had been implicated in these phenotypes through genetic or biochemical evidence [12,13,15]. This was a practical approach prior to the development of genome-wide techniques.

In some instances, DNA methylation changes were observed to persist after cessation of exposure and associated with altered gene expression of nearby phenotype-associated genes. For example, hypomethylation and increased transcript levels of genes implicated in blood pressure regulation in specific tissues: angiotensin II receptor (AT1b) gene in the adrenal gland [15], glucocorticoid receptor (GR) and peroxisome proliferator activated receptor α (PPARα) genes in the liver [12,13] of offspring exposed to maternal protein restriction. In other cases, the changes disappeared after cessation of exposure or had no effect on gene expression. For example, persistent hypermethylation at the promoter of proopiomelanocortin (POMC) gene induced by maternal high-fat diet was detected at both weaning and adult age in the hypothalamus. However, no detectable changes in POMC expression were apparent at either age [20,21].

These early studies demonstrated the utility of using developmental programming as a model for examining the environmental sensitivity of DNA methylation. However, the diversity of animal models, tissues examined, targeted single gene-approaches and techniques for detection of DNA methylation have meant that very few findings have been independently replicated [11,29].

## Challenges with establishing and interpreting environmental influences on DNA methylation

The power to identify DNA methylation changes associated with an environmental exposure was vastly increased by the advent of genome-wide approaches. Quantitative, single-nucleotide resolution of DNA methylation can be achieved using bisulfite treatment, which alters the base pairing properties of the DNA template based on methylation status [30]. This permits the methylation at particular site(s) to be assayed using an array or in a non-targeted manner by sequencing [31–33]. Genome-wide profiling of DNA methylation confirmed the existence and verified the extent to which the distribution of DNA methylation across the genome is influenced by both interindividual genetic variation and the specific cell type.

Large scale multi-omic studies using relatively pure cell types suggest that more than 50% of the epigenetic associations with gene expression can be explained by the presence of common genetic variants occurring in *cis* [34]. Common genetic variants can also influence DNA methylation in *trans*, and rare variants may act similarly [35]. Furthermore, this genetically driven epigenetic variation can be cell-type specific. These findings are important when considering the genetic makeup of the population used for studying environmental effects on DNA methylation, as well as the number of samples required for an adequately powered study. Yet, they also highlight the utility of integrating multiple genome-wide datasets looking at gene expression, genetic variation and DNA methylation to understand how environmentally directed changes in DNA methylation are associated with changes in gene transcription.

Environmental perturbations will also be likely to have cell type specific effects on DNA methylation [36]. This has a number of implications, such as how to identify the relevant cell type and how to interpret data that are usually derived from a tissue biopsy and therefore composed of multiple different cell types [37]. While bioinformatic tools have been developed to infer blood cell composition in humans post-hoc to DNA methylation analysis, such tools are not necessarily available for all cell types or non-human species [38,39].

In the context of developmental programming, environmental insults occur prior to or during cellular differentiation. Since metastable epialleles establish their epigenetic state and maintain it through cellular differentiation, these genomic regions may retain any influence of the environment on DNA methylation in a multi-lineage manner into adulthood [4]. Yet, for genomic regions that are dynamically regulated in development, any environmental legacy on DNA methylation may be restricted to genomic regions not involved in lineage specification for a given cell type [40,41]. A recent study suggests that across tissues derived from the three germ layer lineages (endoderm, mesoderm and ectoderm), and that only 0.1% of the genome shows inter-tissue covariation in the DNA methylation profile [42]. Therefore, it is likely that DNA methylation changes associated with environmental exposures in early development (and throughout life) are cell type restrictive, yet may be functionally relevant.

It is often thought that adult phenotypes associated with developmental programming are due to lasting changes to the epigenome induced by the environment. Yet, the mechanistic evidence is sparse. A transiently imprinted locus provides a good example of how a developmentally regulated transient event can establish an epigenetic state that can have effects in later life. Although not environmentally determined, this might provide a framework for understanding how early environmental insults could have latent effects. The *Gpr1/Zdbf2* locus retains maternal-specific DNA methylation and paternal-specific expression of the *Liz* transcript up unto embryo implantation. This transient expression is sufficient and necessary to establish a permissive epigenetic environment for the later expression of *Zdbf2*, an important gene for brain areas involved in growth regulation and energy homeostasis [43]. In sensitive genomic regions, might environmentally induced transient events during early development potentially act through a similar mechanism?

**Figure 2:**
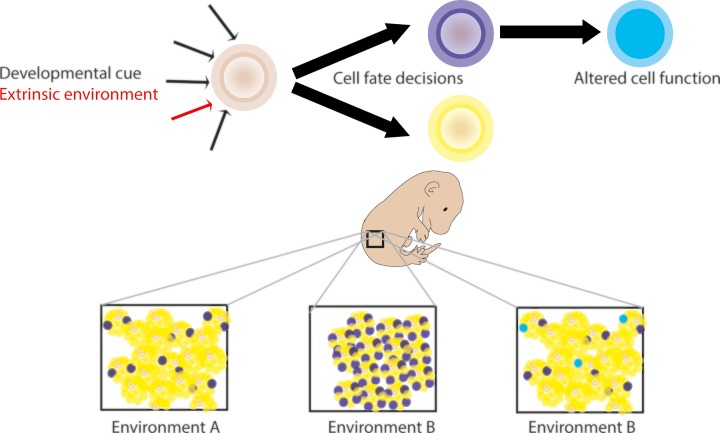
Bulk DNA methylation analysis of tissues can be representative of diverse biological phenomena Cell fate decisions in development are determined by spatially and temporally determined developmental cues. It is possible that cell fate decisions could be perturbed by the developmental environment. This may influence the DNA methylation profile in affected tissues due to a change in the cellular composition (left and middle panels). Alternatively, environmentally induced transcriptional perturbations in development could alter the epigenome in *cis*. In some cells, the changes might be stably maintained through subsequent differentiation and alter the permissiveness for gene expression in specific cell types, conferring altered cell function (left and right panels).

However, changes in DNA methylation do not necessarily imply a causal relationship to the associated phenotype and may be a biomarker of altered physiology [44]. Altered methylation profiles can also reflect changes in the cellular composition of a tissue due to an altered developmental trajectory directed by the environment [45]. While both changes in tissue composition and cell-intrinsic epigenetic perturbations are interesting, it is important to be able to distinguish these in order to improve the understanding of how environmentally associated DNA methylation mechanistically relates to phenotype (Figure 2).

Fortunately, future efforts to address this issue are supported by initiatives to progressively establish reference epigenomes for specific cell types [46,47]. Such references will facilitate the development of bioinformatic pipelines to assess cellular composition of a tissue sample, similar to what is now routine for DNA methylation analyses using peripheral blood in humans. Furthermore, the adaptation of DNA methylation and transcriptome profiling to single-cell analyses provides promise for being able to identify the cell specificity of perturbations in DNA methylation associated with an environmental exposure after disaggregation of tissues [48,49]. Integrating multiple layers of information at the single cell level will establish a platform from which to probe the functional consequences for environmentally induced changes in the epigenome in a targeted manner [50].

Addressing these issues is necessary to be able to further investigate functional changes that may be associated with a detected DNA methylation change. DNA methylation changes associated with a developmental exposure may be useful biomarkers of small changes in the cellular composition of a tissue and provide insight into an associated pathological mechanism. Altered cell-intrinsic methylation can be either causative or consequential of transcriptomic changes associated with the exposure or linked phenotype. Exciting advances in utilising catalytically deactivated nucleases that can be specifically targeted (e.g. CRISPR) to recruit epigenetic modifiers to a specific locus are starting to demonstrate how DNA methylation interacts with other chromatin modifications and is paving the way to be able to address how DNA methylation changes observed in response to an environmental exposure are functionally associated with gene transcription [51,52].

## The consensus so far

There is still a long way to go for the field of environmental epigenetics to move beyond simply establishing an association between an environmental exposure and altered DNA methylation at specific genomic regions. However, some consensus has emerged regarding the characteristics of genomic regions with properties of ‘metastable epialleles’ [4]. That is, genomic regions that show covariation in epigenetic state across tissues derived from all three germ layers.

The prototypical environmentally sensitive metastable epialleles described in mice were both associated with endogenous retroviruses of the Intracisternal A-Particle class, suggesting that there is a genetic component to regions that show pan-tissue interindividual epigenetic variation and sensitivity to the developmental environment [53,54]. However, more recent studies have shown that although these elements make up a significant portion of the mouse genome, most do not behave as metastable epialleles and those that do tend to be evolutionarily young, flanked by methylation-sensitive CTCF-binding sites, and not overlapping annotated transcripts [55,56].

Parallel observations were made in humans, including a lack of overlap with transcribed regions, yet enrichment for regulatory regions like transcriptional start sites and enhancers as well as zinc finger proteins and specific classes of human endogenous retroviruses [57]. In the same study, the methylation dynamics of the identified human metastable epialleles were compared to genome background at multiple stages of early embryonic development, the period in which such regions are hypothesised to be susceptible to environmental perturbation of DNA methylation [58]. In general, the level of DNA methylation in the parental germline and throughout preimplantation development was lower in these regions and remained at an intermediate level in post-implantation foetal tissue whereas generally, upon cellular differentiation a particular CpG site/region usually becomes fully methylated or unmethylated [57]. This indicates that these regions are more variable among cells in the sampled population and this is supported by the within molecule homogeneity observed, suggesting that there is a stochastic component to the establishment of the methylation state for these genomic regions. Interestingly, sites of methylation previously identified to be influenced by the season of conception (and therefore maternal diet) within the rural Gambian population were queried in the methylation profiles from early development and similar methylation dynamics were observed as for the metastable epialleles defined on pan-tissue interindividual variation [59,60].

Although the majority of transposable elements may not behave as metastable epialleles, they still provide good potential candidates for environmentally driven epigenetic effects that might be restricted to particular cell lineages. Transposable elements can act as regulatory elements such as enhancers or promoters to influence the expression of endogenous genes in a developmentally regulated manner [61]. Furthermore, activation of transposable elements in response to environmental exposures is a conserved mechanism across taxa [62]. Outside of the potential to cause genetic variation through transposition, it is tempting to speculate that stress-induced transposable element activation during development could contribute to cell fate decisions and altered tissue formation. Another possible consequence of transient stress-induced transposon expression could be the establishment of an alternative epigenetic state. Such a mechanism could behave similarly to the transient imprinted expression of *Liz* described above to establish a chromatin environment that changes the permissiveness for expression of certain endogenous genes in specific tissues later in development. With improvements in the capacity to distinguish transposable elements at the level of a single integration event, it will be interesting to discover how they contribute both to inter-individual genetic variation and as targets for environmentally driven epigenetic effects [63].

## Off the map

Interestingly, a number of these genome-wide studies also identify an enrichment of metastable epialleles in subtelomeric regions [42,60,64]. Subtelomeric regions are poorly characterised regions in genome assemblies due to the high level of segmental duplications and repetitive sequence that make assembly ambiguous [65]. However, they do contain actively transcribed genes, are CpG dense, highly polymorphic between individuals and demonstrate copy number variation [66,67]. Interestingly, studies in yeast suggest that genes within subtelomeric regions are enriched for functions that are required in environmental interactions, including nutrient transport and metabolism [68]. The increased recombination rates of these regions are thought to rapidly diversify across the yeast population, increasing the probability of some individuals surviving in the event of drastic environmental change [69]. High interindividual genetic variation of these regions is also observed in mammals [66].

Similarly, ribosomal DNA (rDNA) is another highly repetitive region that is also excluded from genome assemblies because of ambiguous mapping [70–72]. rDNA encodes for an indispensable structural and catalytic component of the ribosome, ribosomal RNA (rRNA). Ribosome production is the most energy consuming cellular process and regulation of rRNA transcription is responsive to pathways modulated by nutrient availability and intracellular energy status [73,74]. Recently, it has been shown that DNA methylation of rDNA is altered in offspring of dams fed a low protein diet [75,76]. The diet-induced DNA methylation response was observed in tissues of both endodermal (liver) and ectodermal (sperm) origin. It was also restricted to a subtype of rDNA that could be distinguished by a genetic polymorphism, with the extent of both the methylation response and diet-induced growth restriction proportional to the relative abundance of this subtype within the genome of the individual. Reanalysis of published data found similar results in alternative dietary models of developmental programming [76]. These studies identify rDNA as a potential metastable epiallele that can be influenced by the developmental environment. Like subtelomeric repeats, inter-individual variation within rDNA arises at much higher rates than the genome-wide average, as it acts as a hot-spot for meiotic recombination [77]. Intriguingly, rDNA copy number variation can be regulated by nutrient availability in yeast [78].

Subtelomeric regions and rDNA share typical characteristics of metastable epialleles, multi-lineage covariation in DNA methylation and environmental responsiveness. They also both undergo a higher rate of recombination and demonstrate increased inter-individual genetic variation [77,79,80]. Efforts are being made to map the so called ‘dark matter’ of mammalian genomes, inclusive of these and other highly repetitive regions [81]. To this end utilising a combined approach of both long and short read sequencing provides promise of providing a more inclusive reference genome [82]. It will be intriguing once these technical challenges have been resolved to look into how genetic and epigenetic variation at these regions interact and how both genetic and epigenetic regulation of these regions is responsive to environmental influences.

## Conclusion

DNA methylation has been widely studied in the context of gene–environment interactions. It is well accepted that environmental factors can be associated with altered DNA methylation at specific genomic regions. We have focused on the developmental environment as it builds on the early work utilising metastable epialleles as examples of environmentally sensitive epigenetically driven phenotypes, but it is worthy to note that many other environmental variables have also been shown to associate with replicable changes in DNA methylation (e.g. tobacco smoking) [83].

Despite the substantial research focus on environmental modulation of DNA methylation as a potential mediator of gene–environment interactions, many questions remain. Circumventing the cell-type specificity of the epigenome to identify changes that do not conform to the criteria of metastable epialleles but may be functionally relevant will be challenging. Establishing the functional origin and consequence of DNA methylation associations with environmental exposure will provide great biological insight, but the tools are still in a development phase. Finally, recent evidence suggests that the current genome assemblies may be missing many relevant sites for environmental modulation of epigenetics and that there may be some significance of having a high rate of genetic variation in these regions as well as epigenetic sensitivity to environmental stimuli. Recent technological developments hold the promise to address many of these issues leading to exciting discoveries at the crossroads of gene and environment interactions.

## Summary

- DNA methylation is crucial for normal development in mammals and can be influenced by the environment. However, the extent to which DNA methylation and other epigenetic mechanisms contribute to mediating gene–environment interactions is still unknown.
- Metastable epialleles are rare genomic regions that have their epigenetic state established in early development in a variable manner. Once established the epigenetic state at these regions is stably inherited through subsequent mitotic events. This makes such regions useful for studying the effects of the developmental environment on DNA methylation patterns.
- Genome assemblies are not complete and repetitive regions of mammalian genomes are difficult to study. Recent evidence suggests that the DNA methylation state of these regions may be environmentally sensitive.

## Abbreviations

POMC: proopiomelanocortin
PPARα: proliferator activated receptor α
rDNA: ribosomal DNA
rRNA: ribosomal RNA
